# Calibration Method of a Wideband AC Resistance Voltage Divider Based on an Equivalent Model

**DOI:** 10.3390/s23167181

**Published:** 2023-08-15

**Authors:** Bo Li, Yanping He, Lei Wang, Min Cao, Zhihong Fu, Huiyuan Zhang

**Affiliations:** 1 School of Electrical Engineering, Chongqing University, Chongqing 400044, China; flymicheal@163.com; 2 Electric Power Research Institute of Yunnan Power Grid Co. Ltd., Kunming 650217, China; caomin@dlyjy.yn.csg.cn; 3 Guangzhou GENY Electric Co., Ltd., Guangzhou 510500, China; hsmmxf@163.com; 4 National Institute of Metrology, Beijing 100029, China; kehufuwu@nim.ac.cn; 5 School of Electrical Engineering, Beijing Jiaotong University, Beijing 100044, China; hyzhanga@bjtu.edu.cn

**Keywords:** AC resistance voltage divider, measurement error, frequency characteristic, phase angle error

## Abstract

Aiming at the problem of large measurement error and phase shift in resistance voltage dividers under high-frequency conditions in the field of power measurement, such as power harmonics, an error correction method is proposed for an alternating current (AC) resistance voltage divider based on the equivalence principle. Firstly, the frequency error model of the AC resistance voltage divider precision is established, and the angle difference of the continuous spectrum ratio difference from 50 Hz to 100 kHz is corrected by determining the shielding structure and the resistance parameters and fine-tuning the shielding potential correction method to reduce the capacities error of the AC resistance voltage divider design precision. At the same time, the performance parameters such as the direction and magnitude of the shielding potential compensation capacitive error are investigated. Finally, the precision voltage divider (the maximum voltage applied is 480 V) calibration experiments verify that the important characteristic judgment factor of the voltage divider is independent of the frequency and the equivalent capacitance value, which effectively solves the phase correction problem of harmonic power measurement.

## 1. Introduction

AC resistance voltage dividers are widely used for various levels of voltage measurement [1,2,3,4,5,6], and because the frequency characteristics of resistance voltage divider ratios are easier to achieve than those of electromagnetic transformers and other voltage divider test methods [7,8,9,10,11,12], precision AC resistance voltage dividers are often used under audio or power harmonic measurement conditions to divide higher voltages into lower voltages for measurement, and corresponding national standards have been introduced [13,14,15,16]. However, due to the growing demand for harmonic power measurements, precision resistive voltage dividers require tight control of phase shifts and reduced measurement errors [17,18,19]. Precision harmonic measurements cover the frequency range from the AC power frequency to the 60th harmonic, i.e., 3 kHz, and audio power measurements up to 100 kHz. Finding methods to reduce the impact of capacitive leakage due to resistive connections, resistance to ground, and shielding, and methods to establish harmonic measurement theory and traceability correction methods have become urgent tasks for electrical measurement workers [14,20,21].

The existing research methods are mainly established by the measurement error and voltage divider structure parameters relationship equation and the use of an equipotential shielding technology design voltage divider structure to reduce the measurement error of the resistance voltage divider. In the literature [9], the influence of pulsation components and spurious parameters on the measurement error of a shielded resistance voltage divider was analyzed, and the error variation curve with the divider parameters was obtained by simulations to reduce the error with the proposed measures. In [10,11], a power thermoelectric comparator and a zero-power factor standard source were used to achieve accurate measurement of the phase error of a resistive voltage divider, and a step-climbing method was proposed to achieve accurate measurement of the phase error of the divider based on the micro potentiometer resistance as a reference standard [12]. The literature [14] used digital sampling combined with the step-climbing method to achieve an accurate measurement of the phase error of a resistive voltage divider. The literature [13,14] has proposed a method for MN-type resistive dividers and their phase error traceability and also proposed a method to achieve accurate measurement of the phase error of dividers with different resistance values based on a four-terminal resistive time constant standard combined with a binary reactance divider, and an experimental analysis of the phase error and linearity error of two digitally sampled voltage signals at different voltages. The main and auxiliary voltage divider branches are used to realize the equipotential shielding, which solves the leakage of the resistor connection to the circuit board and the leakage of the distributed capacitance around the resistor. In addition, the traceability test method is as follows: the resistor AC voltage ratio is traceable to the direct current (DC) voltage ratio, the phase error is traceable to the dual-channel digital voltmeter, and the “step-up” method is adopted [2,16], i.e., from low voltage to high voltage, which has been used in the national frequency harmonics standard. However, the phase error of the voltage divider increases with frequency, making the measurement of harmonic power difficult and the cost of point-by-point error correction with reference standards in various complex harmonic conditions too high. The inductive voltage divider with two-stage and single-stage equipotential is used as a reference standard to cover high voltage measurements in the frequency range from industrial frequency 50 Hz to 100 kHz and to achieve AC traceability to DC by extending the resistor in series with the thermocouple, proposing fixed and moving shielding compensation methods. However, there are problems with the measured voltage phase not being measured due to the intervention of the thermocouple and the mobile shield compensation leakage not being given a quantitative analysis [20,22]. In addition, the resistance voltage divider also has application limitations such as a nonlinear high-frequency response [13] and high cost. The limitations of the use of AC resistance voltage dividers are summarized in Table 1 below.

This research establishes a frequency error model for precision AC resistance voltage dividers to study the direction, magnitude, and other performance parameters of the shielding potential compensation capacitive error, and validates the effectiveness of this research’s method for the phase correction problem of harmonic power measurement by setting the shielding structure and resistance parameters to fine-tune the shielding potential for calibration experiments.

## 2. Precision AC Resistance Voltage Divider Frequency Error Analysis

**Figure 1:**
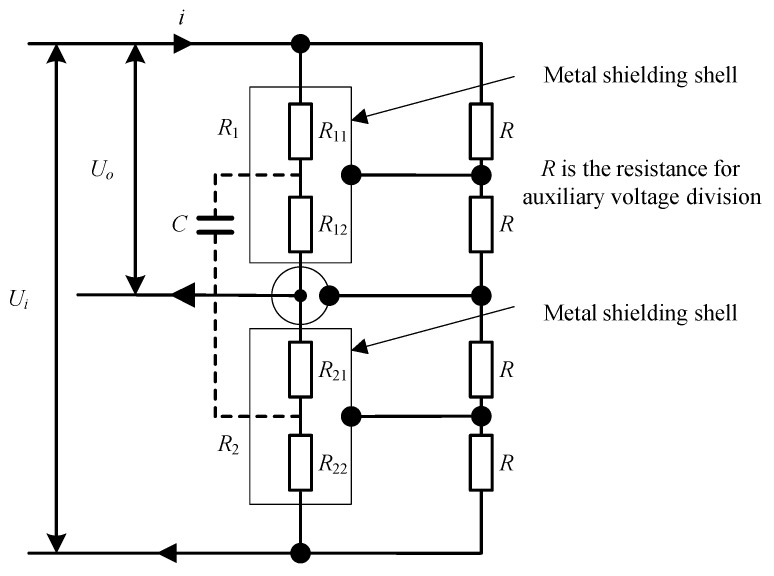
Precision AC resistance voltage divider.

The equivalent circuit topology of the precision AC resistance voltage divider is shown in Figure 1.

As for the parameters in the diagram, *U_i_* and *U_o_* represent input voltage and output voltage, respectively; *R*_1_ represents the voltage divider resistor 1; *R*_2_ represents the voltage divider resistor 2; *C* represents the equivalent leakage capacitance; *R*_11_ represents the equivalent upper resistance of voltage divider resistor 1; *R*_12_ represents the equivalent lower resistance of voltage divider resistor 1; *R*_21_ represents the equivalent upper resistance of voltage divider resistor 2; and *R*_22_ represents the equivalent lower resistance of voltage divider resistor 2.

The electrical structure of the auxiliary voltage divider branch and the equipotential shield are simplified here, while *R*_1_ and *R*_2_ are regarded as ideal (i.e., nominal resistance value, no inductance) single resistors and can be divided into two arbitrary parts that need analysis; usually, the resistance value of R_1_ is several hundred kΩ, the resistance value of R_2_ is several kΩ, the equivalent leakage capacitance *C* is reduced to tens of pF due to the use of equipotential shielding structure, and the above parameters are used as the basic reference for the following error analysis.

(1) $$T=C\times \left(R_{12}+R_{21}\right)$$

(2) $$K=\frac{R_{1}}{R_{1}+R_{2}}$$

(3) ω=2πf

where *T* is the time constant, *K* is the nominal voltage division ratio of the resistive voltage divider, *ω* is the angular frequency (rad), and *f* is the frequency of the voltage divider input signal (Hz).

**Figure 2:**
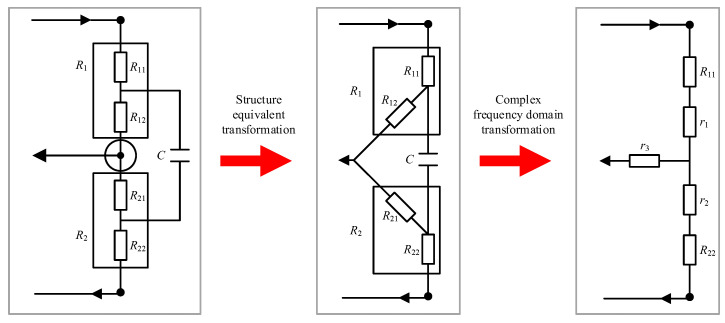
AC resistance voltage divider circuit transformation.

Considering the actual voltage division ratio in the AC case, the equivalent reactance of each branch of the resistance voltage divider is simplified by the ‘Δ-Y’ as shown in Figure 2. The equivalent reactance values r_1_, r_2_ and r_3_ are shown below:

(4) $$r_{1}=\frac{R_{12}}{j\omega C\left(R_{12}+R_{21}\right)+1}$$

(5) $$r_{2}=\frac{R_{21}}{j\omega C\left(R_{12}+R_{21}\right)+1}$$

(6) $$r_{3}=\frac{R_{12}R_{21}}{R_{12}+R_{21}+\frac{1}{j\omega C}}$$

Then, the actual ratio of resistance divider can be obtained and expressed as:

(7) $$K(\omega )=\frac{\omega^{2}T^{2}R_{22}+R_{2}+j\omega TR_{21}}{\omega^{2}T^{2}\left(R_{11}+R_{22}\right)+R_{1}+R_{2}+j\omega T\left(R_{12}+R_{21}\right)}$$

Let *a* = *T*^2^*R*_22_, *b* = *TR*_21_, *c* = *T*^2^(*R*_11_ + *R*_22_), *d* = *T*(*R*_12_ + *R*_21_), then,

(8) $$\begin{array}{ll} K\left(\omega \right) & =\frac{\omega^{2}a+R_{2}+j\omega b}{\omega^{2}c+R_{1}+R_{2}+j\omega d}\\ \; & =\frac{\left(\omega^{2}a+R_{2}\right)\left(\omega^{2}c+R_{1}+R_{2}\right)+\omega^{2}bd+j\left(\omega b\left(\omega^{2}c+R_{1}+R_{2}\right)-\omega d\left(\omega^{2}a+R_{2}\right)\right)}{{\left(\omega^{2}c+R_{1}+R_{2}\right)}^{2}+\omega^{2}d^{2}} \end{array}$$

The value of the imaginary part of the above equation is very small; thus, the contrast calculation can be neglected and the voltage divider ratio *f_c_* is expressed as:

(9) fc(ω)=Re(K(ω))−KK =[(ω2a+R2)(ω2c+R1+R2)+ω2bd](R1+R2)−[(ω2c+R1+R2)2+ω2d2]R2[(ω2c+R1+R2)2+ω2d2]R2

Neglecting the higher order term errors and substituting a, b, c, and d into the final result and simplifying gives:

(10) $$f_{c}\left(\omega \right)=\frac{-\omega^{2}T^{2}\left(R_{11}R_{21}-R_{12}R_{22}\right)\left(R_{11}+R_{22}\right)}{R_{2}{\left(R_{1}+R_{2}\right)}^{2}}$$

Let *p* = *R*_11_*R*_21_ − *R*_12_*R*_22_, then

(11) $$K_{f}=\frac{-T^{2}p\left(R_{11}+R_{22}\right)}{R_{2}{\left(R_{1}+R_{2}\right)}^{2}}$$

The expression for the angular difference of the voltage divider *δ_c_* can be expressed as:

(12) $$\delta_{c}\left(\omega \right)=\frac{\omega T\left[R_{11}R_{21}-R_{12}R_{22}+\omega^{2}T^{2}\left(R_{11}R_{21}-R_{12}R_{22}\right)\right]}{\left(\omega^{2}T^{2}R_{22}+R_{2}\right)\left[\omega^{2}T^{2}\left(R_{11}+R_{22}\right)+R_{1}+R_{2}\right]+\omega^{2}T^{2}R_{21}\left(R_{12}+R_{21}\right)}$$

Ignoring the higher order term errors and substituting a, b, c, and d into the final result and simplifying, the equation can be expressed as:

(13) $$\delta_{c}\left(\omega \right)=\frac{\omega T\left(R_{11}R_{21}-R_{12}R_{22}\right)}{R_{2}\left(R_{1}+R_{2}\right)}$$

Then,

(14) $$K_{t}=\frac{Tp}{R_{2}\left(R_{1}+R_{2}\right)}$$

As a result, the error in the voltage divider can be expressed separately and parsimoniously as:

1. The ratio difference of the voltage divider:

(15) $$f_{c}\left(\omega_{i}\right)=\omega_{i}^{2}K_{f}$$

2. The angle difference of the voltage divider:

(16) $$\delta_{c}\left(\omega_{i}\right)=\omega_{i}K_{t}$$

where the measure of *K_f_* is in seconds squared (*s*^2^) and the measure of *K_t_* is in seconds (*s*), which can be expressed as:

(17) $$K_{f}=\frac{-T^{2}p\left(R_{11}+R_{22}\right)}{R_{2}{\left(R_{1}+R_{2}\right)}^{2}}$$

(18) $$K_{t}=\frac{Tp}{R_{2}\left(R_{1}+R_{2}\right)}$$

The value of *K_f_* is about 1 × 10^−14^, which is determined by the resistance parameters, shielding structure parameters, air medium, etc. It is a constant that does not change with frequency. The agreement between the calculation of the simple Equations (15) and (16), and the exact Equation (7) is about 1 × 10^−20^ in the range of 50 Hz~3 kHz.

The value of *K_t_* is about 1 × 10^−9^, which is determined by the resistance parameters, shielding structure parameters, air medium, etc. It is a constant that does not vary with frequency, and the agreement between the simple Equations (15) and (16), and the calculation of the exact Equation (7) is about 1 × 10^−14^ in the range of 50 Hz~3 kHz.

In this way, the error function at any frequency point in the frequency range measured by the voltage divider can be simplified by its structural characteristic parameters *K_t_* and *K_f_* as:

(19) $$\frac{K\left(\omega_{i}\right)-K}{K}=\omega_{i}^{2}K_{f}+j\omega_{i}K_{t}$$

(20) $$f_{c}\left(\omega_{i}\right)=\omega_{i}^{2}K_{f}$$

(21) $$\delta_{c}\left(\omega_{i}\right)=\omega_{i}K_{t}$$

The first term in Equation (19) is the voltage divider ratio difference and the second term is the voltage divider angle difference. The exact ratio and angle difference of any frequency point in the measurement frequency range is denoted by *f_c_*(*ω_i_*) and *δ_c_*(*ω_i_*), and the frequency point is denoted by *ω_i_*.

The expression for *K_f_* and *K_t_* includes an implicit important judgment factor p, which can be determined from Equations (17), (18), (20), and (21):

When *p* = 0, regardless of how the frequency and equivalent capacitance change, the ratio error and phase difference of the voltage divider are both zero.

When *p* < 0, the ratio error of the voltage divider is positive and its absolute value increases with frequency according to a ω^2^ relationship. The phase difference is negative and its absolute value increases with frequency according to a ω relationship.

When *p* > 0, the ratio error of the voltage divider is negative and its absolute value increases with frequency according to a ω^2^ relationship. The phase difference is positive and its absolute value increases with frequency according to a ω relationship.

*K_t_* and *K_f_* are only related to the structural parameters, and the error caused by changes in signal frequency can be ignored, making them features with frequency invariance. By matching the parameters to make the characteristic factor p close to zero, the characteristic quantities *K_f_* and *K_t_* can be obtained simply by measuring the ratio error and phase difference at any frequency point under a reference standard, as described in Equations (20) and (21). Therefore, the calibration of the voltage divider over the entire frequency range can be completed by performing a source calibration at a single frequency point.

## 3. Adjustment of The Voltage Divider’s Shielding Potential Method for Error Compensation Solutions

By the above analysis of the frequency error of the voltage divider, it can be concluded that the optimal compensation of the frequency error of the voltage divider can be achieved using the adjustment of the shielding potential to the equipotential shield.

**Figure 3:**
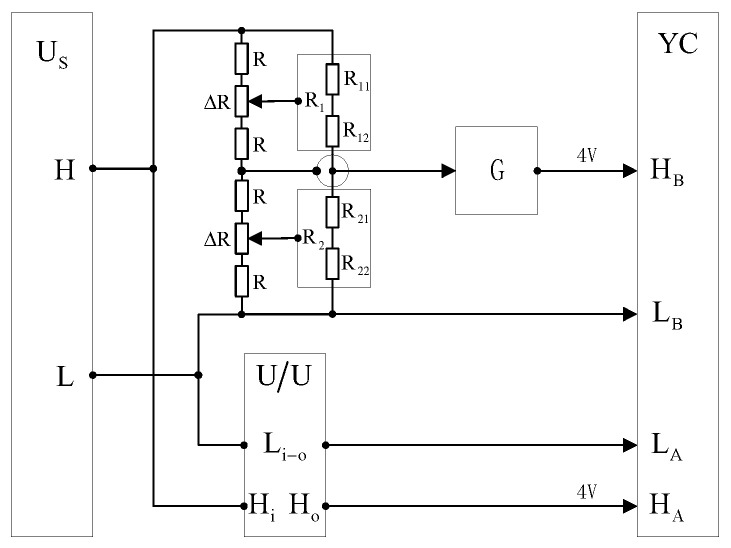
Adjusting the shielding potential diagram of the divider.

Figure 3 shows that ΔR represents the amount of adjustment of the segment resistance R of the auxiliary voltage divider branch. When ΔR is positive, so that the segment resistance increases, the relative potential of the resistive outer shield shell at the corresponding position of the main voltage divider branch shifts down; when ΔR is negative, so that the segment resistance decreases, the relative potential of the resistive outer shield shell at the corresponding position of the main voltage divider rises; G represents the buffer impedance conversion by a precision op-amp; U/U represents the inductive voltage divider for 100 V/4 V equipotential shielding, used as an AC voltage proportional reference standard; Us represents the precision regulated frequency modulation (FM) power supply; and YC represents the dual-channel precision voltage comparator. It should be noted that to ensure the reliability of the comparison, i.e., that the reference standard YC has a high accuracy and stability, the two channels of YC here use the same circuit and electrical isolation design. Among them, a 16-bit analog-to-digital converter AD677 is used for single channel digital acquisition, which has the characteristics of a high precision and a self-calibration function. In terms of the data acquisition control and algorithm, YC can realize the precise measurement of AC voltage based on non-integral period sampling theory, a linear phase algorithm of 90-degree filter window, and real-time polynomial data reconstruction of FFT. The calibration results compared with the national benchmark of the National Institute of Metrology (NIM) in Beijing and the National Research Council of Canada (NRC) show a maximum error of 5 × 10^−6^. Meanwhile, using Fluke732B as a reference, long-term stability and uncertainty analysis tests were carried out from 2019-01 to 2019-10, and the results showed that the measurement extended uncertainty was 1 × 10^−6^.

(1) The principle of equipotential shielding. As shown in Figure 3, for each series voltage divider resistor under the condition of applied voltage, each section of the main and auxiliary voltage divider will produce an equal segment voltage. The single main voltage divider segment resistance will produce the leakage of distributed capacitance due to the connection lines and resistor core to the shielding shell to the ground. The effect of the high end of the segment resistance to the shielding shell at the 1/2 segment potential is that the leakage current flows out from the core and the connecting line, whereas the effect of the low end of the segment resistance to the shielding shell at the 1/2 segment potential is that the leakage current flows in from the core and the connecting line. Therefore, the shielding effect will cancel out the leakage current flowing in, thus eliminating the effect of the distributed capacitance on the measurement. However, the resistor core and connecting line and the axis of the shielding shell are not exactly coincident. The main and auxiliary voltage division of the segment resistance will also have a small difference, which will produce residual distribution capacitance in each main voltage division segment resistance. Its synthetic distribution parameters are shown in Figure 1 and it is the equivalent residual distribution capacitance C which caused the main error of the voltage divider frequency measurement.
(2) The principle of distribution parameters elimination. The elimination of the residual distribution capacitance type is difficult to solve as a process problem. The method chosen in this research is to use the adjustment of the shielding potential, that is, to change the 1/2 section potential equivalent in order to change the proportion of leakage current outflow and inflow. This is performed to achieve the equivalent voltage division on the resistance and the lower resistance and the equivalent capacitance position, so that the judgment factor *p* tends to the minimum value.
(3) Adjustment method. The low end of the precision-regulated FM power supply is connected to the low end of the 100 V/4 V divider, the low end of the 100 V/4 V inductive divider, and the low end of the dual channel voltage comparator A and B channels. The high end of the precision voltage regulator is connected to the high end of the voltage divider and the high end of the inductive divider. The high end of channel A of the dual channel comparator is connected to the low voltage 4 V tap of the inductive divider, and the 4 V tap of the divider is connected to the high end of channel B of the voltage comparator via a buffer.

From a precision-regulated FM power supply output of 1000 Hz 100 V voltage, a dual-channel voltage value can be obtained from the voltage divider proportional error and angular error. Here is an example of the converter angle difference conversion: the indicated value of 0.005°, according to Equation (21), can be calculated to obtain *K_t_* = 1.39 × 10^−8^.

At this point in the voltage divider judgment factor *p* > 0 situation, it is necessary to adjust the shielding potential to bring *K_t_* to zero, that is, equivalently ΔR increases in the positive direction, making *K_t_* or *p* move in the direction of zero, and adjusting to zero. At this point, the error of the voltage divider is compensated.

To review the calibration results, the stable power supply outputs 100 V at 500 Hz and 200 Hz, respectively, and the difference between the angular indications of the two channels should be constant at zero.

The above adjustments are already sufficient for the calibration of frequency errors from 50 Hz to 3 kHz so that the calibration of harmonics is thus fully calibrated and no further digital calibration is required.

## 4. Digital Calibration of Frequency Errors in Harmonics and Audio

In cases where the shielding potential cannot be adjusted for structural reasons, a simple digital correction method can be adopted, which is exactly equivalent to the hardware adjustment of the shielding potential. Regardless of single frequency or complex frequency conditions, this research can be used to obtain the error of the resistive voltage divider at any frequency point *ω_i_*. *i* is the number of harmonics 1, 2, ……, 60 representing 50, 100, ……, 3000 Hz frequency points, respectively.

To illustrate: the abovementioned *K_t_* = 1.39 × 10^−8^, the errors at 200 Hz, 500 Hz, 1000 Hz, and 3000 Hz are calculated according to Equation (21) as: ***δ***_4_ = 17.5 × 10^−6^ rad, ***δ***_10_ = 43.6 × 10^−6^ rad, ***δ***_20_ = 87.3 × 10^−6^ rad, ***δ***_60_ = 261.8 × 10^−6^ rad.

In the case of audio, the above calculation method is also used, provided that the *K_t_* value is appropriate for any frequency point in the measurement range, e.g., 1001.11 Hz, which can also be calculated accurately according to the above equation.

## 5. Experimental Data Validation to Eliminate Voltage Divider Errors

By measuring the angular differences of six voltage dividers described in the literature [2,16], the data shown in Table 2 were obtained. Here, the angular differences of the six voltage dividers are measured by the step-up method using the China National Industrial Frequency Harmonic Measuring Device of the National Institute of Metrology in Beijing, China. Fluke6100A is used as a signal source, and two digital voltmeters (HP3458) are used as sampling ADCs. Then, the angle difference between the two voltage signals can be calculated by synchronous sampling compensation and discrete Fourier transformation (DFT) data processing. The DFT used a total of 1680 sampled data in four cycles.

The *K_t_* values were calculated using Equation (21) for the data in Table 2 and the results are shown in Table 3.

Considering that the resistance voltage divider needs to focus on the frequency band of 1 kHz and above, only the statistical analysis of *K_t_* value at the frequency band of 1 kHz and above is conducted. The results are shown in Table 4. In fact, the calculated *K_t_* value in the frequency range of 50 Hz~1 kHz still has the same order of magnitude.

According to the method described in this article, error correction was performed on two sets of resistor dividers, 120 V/0.8 V and 480 V/0.8 V, with correction parameters of R_1_ = 298 KΩ, R_2_ = 2 KΩ, equivalent capacitance of 30 pF, R_12_ = 0, R_21_ = 1735 Ω, R_22_ = 265 Ω, and *K_t_* = 4.49 × 10^−9^, and R_1_ = 599 KΩ, R_2_ = 1 KΩ, equivalent capacitance of 10 pF, R_12_ = 54 KΩ, R_21_ = 0, R_22_ = 1000 Ω, and *K_t_* = −4.86 × 10^−8^. The phase difference and actual measurement error at each frequency point were calculated and compared with the actual test values before correction, as shown in Table 5 and Table 6. From the table, it can be seen that the actual measured data with the angular difference (Table 2) exactly match, with only a small deviation from the literature [2] step-up phase error traceability process.

Based on the data in Table 5 and Table 6, further optimization parameter matching and calibration were carried out for 120 V/0.8 V and 480 V/0.8 V. The parameters used were an equivalent capacitance of 30 pF, R_12_ = 1000 Ω, R_21_ = 10 Ω, R_22_ = 1990 Ω, and *K*_t_ = 4.95 × 10^−11^ for 120 V/0.8 V, and an equivalent capacitance of 10 pF, R_12_ = 54 KΩ, R_21_ = 90 Ω, R_22_ = 910 Ω, and *K*_t_ = −8.11 × 10^−11^ for 480 V/0.8 V. The phase difference and actual measurement error at each frequency point were calculated and compared with the actual test values before calibration. The results are shown in Table 7 and Table 8.

From the data in the table, it can be seen that after fine calibration of the divider using the method described in this paper, the maximum angle difference of the divider calculated using the *K*_t_ value obtained is no more than 2 × 10^−6^ rad within the frequency range of 50 Hz to 3 kHz. The actual tested and calibrated angle difference of the divider also matches the calculated value very closely, indicating that the model method is correct and reliable. Moreover, in terms of the angle difference over the entire frequency range, the difference between each frequency point is particularly small, almost independent of the frequency size. In general harmonic power testing applications, the angle difference of the divider can be completely ignored, and there is no need for digital compensation. Comparing the measured data before and after calibration, the angle difference of the calibrated divider is only 1/(500 to 900) of the angle difference before calibration, and the error level has increased by about 2 to 3 orders of magnitude. Comparing the angle difference data of the two dividers in Table 7 and Table 8, it can be seen that the angle difference of the divider with a higher operating voltage is larger, indicating that there are different leaks in the selection, structure, and processing technology of the divider device at different voltage levels, and it is necessary to further study and improve the performance indicators.

**Figure 4:**
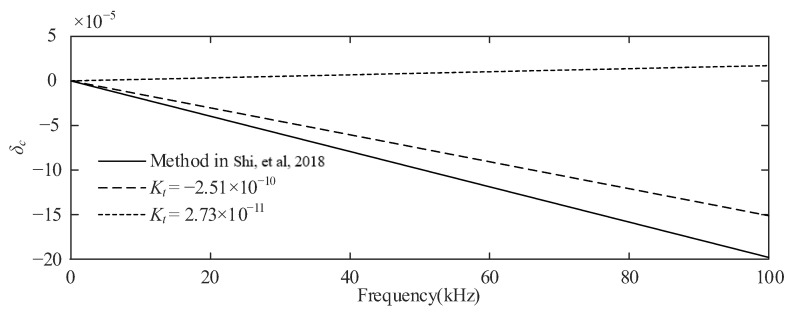
Comparison of angular difference measurements [13].

Since the upper limit of the test frequency in the literature [2,14] is 3 kHz, the test results in the literature [13] are used to verify the validity of the correction method when the frequency range is greater than 3 kHz. The phase angle error of the resistive voltage divider, the ratio of which is 100:1 with 11 sets of 100 Ω resistive elements in serial connection and 9 sets of 100 Ω resistive elements, is still designed by the National Institute of Metrology in Beijing and measured at the voltage of 30 V and shown in Figure 8 of the literature [13]. According to Equation (6) in [13], after the leakage capacitance determined by the geometry and resistance are determined, the phase angle errors present a linear relationship with the frequency, which is also consistent with the results of Equation (21) in this paper. According to the phase angle error test results shown in Figure 8 and Equation (21), we can obtain an estimate of *K_t_* of about −3.15 × 10^−10^. Then, we still assume that the equivalent capacitance is 30 pF, and two sets of resistance values, (R_11_ = 1000 Ω, R_12_ = 100 Ω, R_21_ = 1/9 Ω, R_22_ = 99/9 Ω) and (R_11_ = 1000 Ω, R_12_ = 100 Ω, R_21_ = 10/9 Ω, R_22_ = 90/9 Ω), respectively, are adopted according to the circuit topology and the ratio K = 100:1 in [13]. From Equations (1) and (18), the corresponding *K_t_* of the two sets of resistance values are −2.51 × 10^−10^ and 2.73 × 10^−11^, respectively. The measurement results of the resistive voltage divider are shown in Figure 4 where the solid line represents the method of the literature [13] and the dashed line represents the method of this paper. The figure shows that the frequency response of the correction method proposed in this paper is still better in the frequency range of 3 kHz to 100 kHz. In addition, it should be noted that when a higher voltage is applied, the temperature increase and possible corona discharge will amplify the error of the voltage divider. The error compensation correction method proposed in this paper is limited to a resistance voltage divider below 480 V.

## 6. Design and Calibration Steps for Precision Voltage Dividers

The suitable appropriate shielding structure and R_1_ and R_2_ parameters are selected by experimentation so that the judgment factor *p*-value is as small as possible. For example, in [5], the 480 V voltage divider R_1_ = 599 kΩ, R_2_ = 1 kΩ, *K_t_* value is negative, the 240 V voltage divider R_1_ = 598 kΩ, R_2_ = 2 kΩ, *K_t_* value is positive, and the appropriate R_1_, R_2_ are selected to obtain a smaller *K_t_* value design. The *K_t_* value is taken as the adjustment target because, usually, *K_f_* value is 4–5 orders of magnitude smaller than the *K_t_* value. As long as the *K_t_* value is selected, a smaller *K_f_* value will be obtained due to the small *p*-value.

Once the shielding structure and resistance parameters have been selected, the resistance of the auxiliary branch is fine-tuned so that the shielding potential moves in the direction of a reduced *p*-value. When the *p*-value is positive, move up; when the *p*-value is negative, move down.

Once the above two steps have been completed, or if for other reasons it is not possible to select another resistor parameter and shielding structure, the voltage divider can be digitally corrected.

**Figure 5:**
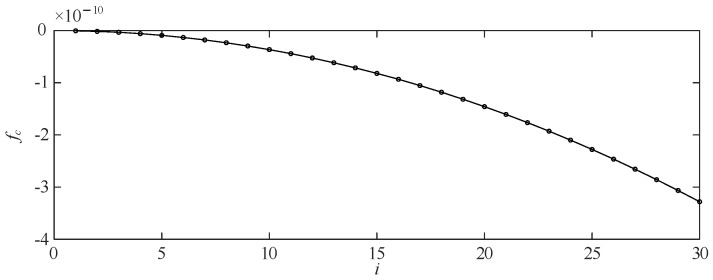
The ratio difference when adjusting the shielding potential diagram of the divider.

**Figure 6:**
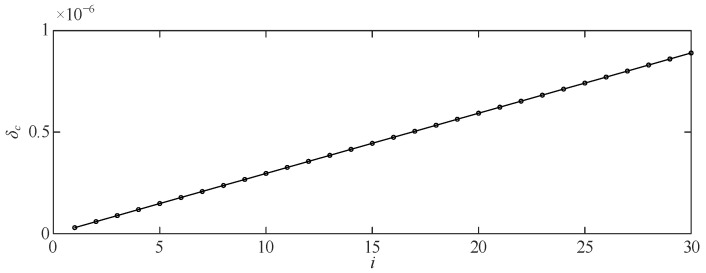
The angle difference when adjusting the shielding potential diagram of the divider.

Regarding the equipotential inductive voltage divider, a suitable frequency point such as 1 kHz is selected, and a selected voltage ratio such as 120 V/0.8 V is measured to obtain the ratio measurement value *f*_c_ and the angular difference measurement value *δ_c_*. *K_f_* and *K_t_* are calculated according to Equations (20) and (21), and the error at any frequency point *ω_i_* (including the calibration point 1 kHz itself) can be calculated by substituting *K_f_* and *K_t_* into Equations (20) and (21) This completes the error calibration of the continuous spectrum from 50 Hz to 100 kHz. Figure 5 and Figure 6 show the relationship between the quadratic characteristic of the signal frequency for the voltage divider ratio difference and the linear relationship for the signal frequency for the angular difference, which has been assumed to be at the optimum compensation matching parameters.

## 7. Conclusions

This research proposes a frequency error model for AC resistance dividers’ precision. In the design of AC resistive voltage dividers precision, capacitive errors caused by various reasons are controlled. A single point correction method is used to achieve the correction of the continuous spectral ratio difference angle difference between 50 Hz and 100 kHz. The direction and magnitude of the shielding potential compensation are investigated for capacitive error. It is proposed that the important characteristic *p*-factor of the divider is independent of the frequency and equivalent capacity value. The phase correction problem for harmonic power measurement is solved.

## Figures and Tables

**Table 1:** The limitations of the use of AC resistance voltage dividers.

Manufacturing Cost	Production Difficulty	Ratio Adjustment	Error Calibration	Output Impedance	Digital Compensation
High	High	Hard (difficult to automate)	Hard (difficult to cover the full frequency band)	High	NO

**Table 2:** Angular difference measurements for six voltage dividers from the literature [2].

*f* (Hz)	*δ* (×10^−6^ rad)					
	480 V	240 V	120 V	60 V	30 V	15 V
50	−14.4	12.2	14.6	13.0	1.1	3.4
500	−150.8	118.2	143.0	131.9	14.2	43.0
1000	−305.7	233.4	282.7	260.3	26.8	83.8
1500	−451.1	356.8	430.2	396.1	45.1	129.1
2000	−614.9	459.8	559.4	515.4	50.6	163.4
2500	−776.7	567.7	694	639.5	66	205.7
3000	−918.4	701.9	851.3	785.9	87.8	250.3

**Table 3:** Calculation of *K_t_* values for six voltage dividers from the literature [2].

*f* (Hz)	*K_t_*(*s*) (×10^−9^)					
	480 V	240 V	120 V	60 V	30 V	15 V
50	−45.9	38.9	46.5	41.4	3.50	10.8
500	−48.0	37.6	45.5	42.0	4.52	13.7
1000	−48.7	37.2	45.0	41.4	4.27	13.3
1500	−47.9	37.9	45.7	42.0	4.79	13.7
2000	−49.0	36.6	44.5	41.0	4.03	13.0
2500	−49.5	36.2	44.2	40.7	4.20	13.1
3000	−48.7	37.3	45.2	41.8	4.66	13.3

**Table 4:** Analysis of calculated *K_t_* values from 1 kHz to 3 kHz.

	*K_t_*(s) (×10^−9^): Mean and Standard Deviation from 1 kHz to 3 kHz					
	480 V	240 V	120 V	60 V	30 V	15 V
Average	−48.8	−37.0	44.9	41.4	43.9	13.3
Std deviation	0.581	0.658	0.589	0.522	0.321	0.268

**Table 5:** Comparison of 120 V/0.8 V calculated angular differences based on *K_t_* values and measured values.

*f* (Hz)	*δ_c_* (×10^−6^ rad)						
	50	500	1000	1500	2000	2500	3000
Calculating angular differences	14.1	141	282	423	563	704	845
Measured angle difference	14.6	143	282.7	430.2	559.4	694	851.3
Difference	−0.5	−2	−0.7	−7.2	3.6	10	−6.3

**Table 6:** Comparison of 480 V/0.8 V calculated angle differences based on *K_t_* values and measured values.

*f* (Hz)	*δ_c_* (×10^−6^ rad)						
	50	500	1000	1500	2000	2500	3000
Calculating angular differences	−15.3	−153	−305	−458	−610	−763	−916
Measured angle difference	−14.4	−150.8	−305.7	−451.1	−614.9	−776.7	−918.4
Difference	−0.9	−2.2	0.7	−6.9	4.9	13.7	2.4

**Table 7:** Comparison of the calculated angular difference of 120 V/0.8 V based on *K_t_* values with the measured values after fine matching.

*f* (Hz)		*δ_c_* (×10^−6^ rad)						
		50	500	1000	1500	2000	2500	3000
Before calibration	Calculating angular differences	0.02	0.16	0.31	0.47	0.62	0.78	0.93
	Measured angle difference	0.02	0.17	0.35	0.52	0.72	0.87	0.96
After calibration	Measured angle difference	14.6	143	282.7	430.2	559.4	694	851.3

**Table 8:** Comparison of the calculated angular difference of 480 V/0.8 V based on *K_t_* values with the measured values after accurate matching.

*f* (Hz)		*δ_c_* (×10^−6^ rad)						
		50	500	1000	1500	2000	2500	3000
Before calibration	Calculating angular differences	−0.03	−0.26	−0.51	−0.76	−1.02	−1.27	−1.53
	Measured angle difference	−0.02	−0.31	−0.52	−0.79	−1.05	−1.31	−1.57
After calibration	Measured angle difference	−14.4	−150.8	−305.7	−451.1	−614.9	−776.7	−918.4

## Data Availability

Not applicable.

## References

[1] Bogarra S., Saura J., Rolán A. (2022). New Smart Sensor for Voltage Unbalance Measurements in Electrical Power Systems. Sensors.

[2] Lei W., Zuliang L., Min L., Lijuan L., Hao Z. A Wide Frequency Resistive Voltage Divider for Harmonic Power at Industrial Frequency. Proceedings of the 2007 4th National Electromagnetic Metrology Conference, (NEMC).

[3] Bing Y., Lin Z., Haofan L., Luyao Z., Zhi Y., Shuang L. Analysis of 500kV Capacitive Voltage Transformer Leakage Fault. Proceedings of the 2021 6th International Conference on Integrated Circuits and Microsystems (ICICM).

[4] Karaman I. Implementation and Analysis of Reference Very Low Frequency (VLF) AC High Voltage Measuring System. Proceedings of the 2019 11th International Conference on Electrical and Electronics Engineering (ELECO).

[5] Zhou Q., He W., Li S., Hou X. (2015). Research and Experiments on a Unipolar Capacitive Voltage Sensor. Sensors.

[6] Small G.W., Budovsky I.F., Gibbes A.M., Fiander J.R. (2005). Precision three-stage 1000 V/50 Hz inductive voltage divider. IEEE Trans. Instrum. Meas..

[7] Solomon A., Mwaniki F.M., Vermeulen H.J. Application of Pseudo-Random Impulse Perturbation for Characterizing Capacitor Voltage Transformer Frequency Responses. Proceedings of the 2020 6th IEEE International Energy Conference (ENERGYCon).

[8] Sakata S., Okamura Y. (2014). Phosphatase activity of the voltage-sensing phosphatase, VSP, shows graded dependence on the extent of activation of the voltage sensor. J. Physiol..

[9] Zheng J., Li B., Zha K., Guo N., Wang L. (2021). Equipotential shielding voltage sensor for contact measurement of transient voltage in EHV/UHV power grids. High Volt..

[10] Krause T.C., Camenzind K., Green D.H., Moeller A., Huchel L., Leeb S.B. (2022). A Sensor Topology for Noncontact AC Voltage Measurement of Polyphase Cables. IEEE Trans. Instrum. Meas..

[11] Lawrence D., Donnal J.S., Leeb S., He Y. (2016). Non-Contact Measurement of Line Voltage. IEEE Sensors J..

[12] Budovsky I., Gibbes A., Arthur D. (1999). A high-frequency thermal power comparator. IEEE Trans. Instrum. Meas..

[13] Shi Z., Zhang J., Pan X., He Q., Lin J. (2018). Self-Calibration of the Phase Angle Errors of RVDs at Frequencies Up to 100 kHz. IEEE Trans. Instrum. Meas..

[14] Shi Z., Zhang J., Pan X., Song Y., Lin J., He Q. (2019). Self-calibration and verification of phase angle errors of two voltage dividers at high frequencies. IEEE Trans. Instrum. Meas..

[15] Pan X., Zhang J., Shi Z., He Q., Lin J. (2018). Establishment of AC power standard at frequencies up to 100 kHz. Measurement.

[16] Lu Z., Wang L., Li M., Liu L., Zhou H. (2010). Harmonic power standard at NIM and its compensation algorithm. IEEE Trans. Instrum. Meas..

[17] Budovsky I., Gibbes A., Hammond G. Voltage Divider Characterization at Frequencies up to 200 KHz. Proceedings of the Conference on Precision Electromagnetic Measurements. Conference Digest. CPEM 2000.

[18] Budovsky I. (2009). Standard of Electrical Power at Frequencies Up to 200 kHz. IEEE Trans. Instrum. Meas..

[19] Budovsky I. (2007). Measurement of Phase Angle Errors of Precision Current Shunts in the Frequency Range From 40 Hz to 200 kHz. IEEE Trans. Instrum. Meas..

[20] Bergsten T., Tarasso V., Rydler K. Precision Measurement System for Characterisation of Phase Displacement of Voltage Dividers up to 1 MHz. Proceedings of the Conference on Precision Electromagnetic Measurements (CPEM 2010)..

[21] Rietveld G., Zhao D., Kramer C., Houtzager E., Kristensen O., de Leffe C., Lippert T. (2011). Characterization of a wideband digitizer for power measurements up to 1 MHz. IEEE Trans. Instrum. Meas..

[22] Pogliano U., Trinchera B., Lanzillotti M., Serazio D. Characterization of resistive dividers for a wideband power analyzer. Proceedings of the 29th Conference on Precision Electromagnetic Measurements (CPEM 2014).

